# Developing Therapies for C3 Glomerulopathy: Report of the Kidney Health Initiative C3 Glomerulopathy Trial Endpoints Work Group

**DOI:** 10.2215/CJN.0000000000000505

**Published:** 2024-06-03

**Authors:** Carla Nester, Dima A. Decker, Matthias Meier, Shakil Aslam, Andrew S. Bomback, Fernando Caravaca-Fontán, Terence H. Cook, David L. Feldman, Veronique Fremeaux-Bacchi, Daniel P. Gale, Ann Gooch, Sally Johnson, Christoph Licht, Mohit Mathur, Matthew C. Pickering, Manuel Praga, Giuseppe Remuzzi, Viknesh Selvarajah, Richard J. Smith, Hossein Tabriziani, Nicole van de Kar, Yaqin Wang, Edwin Wong, Kirtida Mistry, Mark Lim, Cesia Portillo, Seyi Balogun, Howard Trachtman, Aliza Thompson

**Affiliations:** 1 Department of Pediatrics, Division of Nephrology, Carver College of Medicine, University of Iowa, Iowa City, Iowa; 2 Apellis Pharmaceuticals Inc., Waltham, Massachusetts; 3 Novartis Inc., Basel, Switzerland; 4 BioCryst Pharmaceuticals Inc., Durham, North Carolina; 5 Columbia University Irving Medical Center, New York, New York; 6 Research Institute “Hospital 12 de Octubre,” Madrid, Spain; 7 Department of Immunology and Inflammation, Imperial College London, London, United Kingdom; 8 National Kidney Foundation, New York, New York; 9 Hôpital Europeén Georges Pompidou, Paris, France; 10 Department of Renal Medicine, University College of London, London, United Kingdom; 11 Rare Kidney Disease Registry (RaDaR), Bristol, United Kingdom; 12 Great North Children's Hospital, Newcastle upon Tyne, United Kingdom; 13 The Hospital for Sick Children, Toronto, Ontario, Canada; 14 Visterra Inc., Waltham, Massachusetts; 15 Department of Medicine, Nephrology Department, Complutense University, Madrid, Spain; 16 Istituto di Ricerche Farmacologiche Mario Negri IRCCS, Bergamo, Italy; 17 Research and Early Development, Cardiovascular, Renal and Metabolism, Biopharmaceuticals R&D, AstraZeneca, Cambridge, United Kingdom; 18 Molecular Otolaryngology and Renal Research Laboratories, Carver College of Medicine, University of Iowa, Iowa City, Iowa; 19 Natera Inc., Austin, Texas; 20 Radboud Institute for Molecular Life Sciences, Amalia Children's Hospital, Radboud University, Nijmegen, The Netherlands; 21 Novartis Inc., East Hanover, New Jersey; 22 Translational and Clinical Research Institute, Newcastle University, Newcastle Upon Tyne, United Kingdom; 23 Center for the Drug Evaluation and Research, US Food and Drug Administration, Silver Spring, Maryland; 24 Kidney Health Initiative, American Society of Nephrology, Washington, DC; 25 Department of Pediatrics, University of Michigan, Ann Arbor, Michigan

**Keywords:** CKD, clinical nephrology, clinical trial, complement, GN

## Abstract

Randomized clinical trials are underway to evaluate the efficacy of novel agents targeting the alternative complement pathway in patients with C3 glomerulopathy (C3G), a rare glomerular disease. The Kidney Health Initiative convened a panel of experts in C3G to (*1*) assess the data supporting the use of the prespecified trial end points as measures of clinical benefit and (*2*) opine on efficacy findings they would consider compelling as treatment(s) of C3G in native kidneys. Two subpanels of the C3G Trial Endpoints Work Group reviewed the available evidence and uncertainties for the association between the three prespecified end points—(*1*) proteinuria, (*2*) eGFR, and (*3*) histopathology—and anticipated outcomes. The full work group provided feedback on the summaries provided by the subpanels and on what potential treatment effects on the proposed end points they would consider compelling to support evidence of an investigational product's effectiveness for treating C3G. Members of the full work group agreed with the characterization of the data, evidence, and uncertainties, supporting the end points. Given the limitations of the available data, the work group was unable to define a minimum threshold for change in any of the end points that might be considered clinically meaningful. The work group concluded that a favorable treatment effect on all three end points would provide convincing evidence of efficacy in the setting of a therapy that targeted the complement pathway. A therapy might be considered effective in the absence of complete alignment in all three end points if there was meaningful lowering of proteinuria and stabilization or improvement in eGFR. The panel unanimously supported efforts to foster data sharing between academic and industry partners to address the gaps in the current knowledge identified by the review of the end points in the aforementioned trials.

## Background

C3 glomerulopathy (C3G) is a rare primary kidney disorder with an incidence of approximately 1–2 patients/million population per year. It can present with asymptomatic urinary abnormalities, acute nephrotic or nephritic syndromes, and severe AKI.^1–3^ Nearly 50% of patients progress to kidney failure over a 10–15-year follow-up period, and the disease recurs in most of patients with C3G who receive a kidney transplant.^4^ The disease is characterized by dysregulation of the alternative pathway of complement and is diagnosed by finding predominant C3 immunofluorescence staining of the glomeruli and active GN on kidney biopsy and after alternative diagnoses have been eliminated.^4,5^

Patients with C3G are treated with a variety of antiproteinuric, antihypertensive, and immunosuppressive drugs with variable efficacy.^6–8^ While there are currently no approved therapies for C3G, a number of agents that target the complement system at different stages along the inflammatory cascade are being evaluated as treatments of C3G. In 2021, the Kidney Health Initiative (KHI), a public–private partnership between the American Society of Nephrology and the US Food and Drug Administration (FDA), began a project to address some of the challenges associated with developing novel treatments for C3G. The project, as initially envisioned, included three stages. The first stage consisted of a review of the published literature on the natural history of disease and markers that could potentially be used to identify patients at greater risk of disease progression and as efficacy end points in clinical trials of C3G. The second and larger goal of the project was to create a central data repository from observational and interventional trials, which, in the third stage of the project, could be used to support the design of future clinical trials in C3G.

In 2023, leadership in the Division of Cardiology and Nephrology at the FDA requested that the work group shift the short-term goals of the project to address a more immediate need. At the time of the request, there were two phase 3 trials in C3G ongoing, and leadership in the Division of Cardiology and Nephrology sought further discussion of the prespecified end points that were being used in these trials before study completion and release of the results. Specifically, the Division of Cardiology and Nephrology wanted to understand how experts in the disease area viewed these end points, and their impression of the evidence supporting their use, given the limitations of the available data supporting each of the proposed end points, including a lack of information on the magnitude of change that could be considered clinically meaningful. To address this request, KHI convened a panel of key opinion leaders with expertise in C3G to assess the data supporting the use of these end points as measures of clinical benefit and to opine on efficacy findings that the work group might consider to be compelling as treatment(s) of C3G in native kidneys. This study summarizes the C3G Trial Endpoints Work Group's methods, deliberations, and conclusions.

## Methods

In June 2023, KHI reconvened a panel of experts in C3G and launched the C3G Trial Endpoints Work Group. Participants were key opinion leaders in the field and included clinical nephrologists, pathologists, basic scientists, pharmaceutical leaders, patient advocacy groups, and FDA representatives. All meetings were conducted by Zoom.

In the first step of the review, two subgroups were created, each led by an industry sponsor scientist (D.A. Decker and M. Meier), one for each ongoing phase 3 trial. Each subgroup comprised a core team that included three academic scientists and/or clinicians with expertise in the pathogenesis and/or treatment of C3G in native kidneys. The topic of recurrent C3G after transplantation was not part of the work group's deliberations. The subgroups combined included four academic scientists from Europe (E. Wong, M. Praga, T.H. Cook, and D.P. Gale) and two from the United States (R.J. Smith and A.S. Bomback). An attempt was made to ensure that academic reviewers were not asked to assess data that they were instrumental in developing. Each subgroup was charged with defining the key efficacy end points being used in the ongoing trials and to specify what clinical outcomes these end points were expected to predict. They were also tasked with summarizing the available evidence that supported the use of these end points as surrogate measures for the stated clinical outcomes, including both the strengths of the evidence and the weaknesses. Each subgroup met approximately biweekly between July 6 and October 10, 2023. The two subgroups met together twice in September 2023 to reach alignment on the evidence and uncertainties surrounding the trial end points.

In the second step of the project, the findings of the two subgroups were presented to the entire work group on November 17, and November 29, 2023. The findings of the two subgroups regarding the trial end points were presented during the first meeting with an opportunity for work group members to ask questions and to clarify the evidence and uncertainties surrounding the end points as surrogates for clinical outcomes. At a second meeting, work group members were tasked with considering the information presented by the subgroups and opining on what potential treatment effects on the proposed end points they would consider compelling to support evidence of an investigational product's effectiveness for treating C3G.

**Figure 1:**
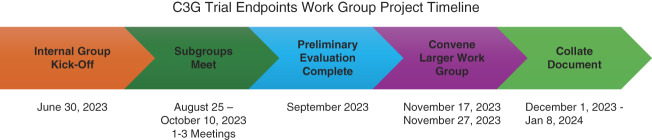
**This schematic illustrates the time line for the activities of the KHI C3G Trials Endpoint Work Group.** C3G, C3 glomerulopathy; KHI, Kidney Health Initiative.

The work group chair (C. Nester), KHI Liaison (H. Trachtman), KHI staff members (C. Portillo, S. Balogun, and M. Lim), and FDA participants (K. Mistry and A. Thompson) served as the steering committee for the project and met monthly to monitor the progress of the project. They were responsible for scheduling meetings, drafting summaries of the proceedings, and compiling a draft manuscript. The report includes key references that highlight the issues surrounding the three prespecified trial end points. A complete list of the citations reviewed by the work group are provided in the Supplemental Appendix 1. The Steering Committee members drafted the manuscript, and the final version of the study was reviewed and approved by all members of the C3G Trial Endpoints Work Group on January 8, 2024. The time line of the project is illustrated in Figure 1.

## Results

### Trial End Points and the Outcomes They Are Intended to Predict

At the time of the initiation of the project, there were two ongoing trials in C3G, one assessing iptacopan, an inhibitor of factor B (Novartis),^9^ and another assessing pegcetacoplan, a direct C3 and C3b inhibitor (Apellis).^10^ The trials included a 6-month double-blind placebo-controlled period, followed by a 6-month open-label extension period during which all patients received the investigational product. Efficacy will be evaluated at the end of the double-blind placebo-controlled period.

Both trials are assessing similar key efficacy end points at 6 months, *i.e*., the effects of the investigational product on proteinuria, eGFR, and histopathology. In both trials, the proteinuria end point assesses the change in urine protein:creatinine ratio (UPCR) from baseline to week 26/month 6 with the anticipation that an effect on such an end point would be associated with the outcome of reduced risk of progression of kidney disease or kidney failure. For the eGFR end point, both trials are assessing the change in kidney function, either stabilization or decrease in the rate of decline, from baseline to week 26/month 6 with the anticipation that an effect on such an end point would also be associated with a lower likelihood of kidney failure or progression of kidney disease. The histopathology end point has two components across the trials. While both trials include similar broad histopathology-based end points (C3c deposition and C3G histologic activity score), the prespecified efficacy end points are somewhat different. The C3c deposition immunohistopathology end point, specifically a reduction in staining intensity as compared with baseline biopsy, was incorporated into both trials to reflect direct attenuation of the underlying disease mechanism. Changes from baseline in the activity score using standard pathology methods, specifically reduction in the C3G activity histopathology score was anticipated to be associated with a reduced likelihood of progression of kidney disease. The end points in the two ongoing trials are summarized in Table 1.

**Table 1:** End points in C3 glomerulopathy trials

End Point	Factor B Inhibitor	C3 Inhibitor
Primary end points	The log-transformed ratio to baseline in UPCR (sampled from a 24-h urine collection) at 6 mo	The log-transformed ratio of UPCR at week 26 compared with baseline
Secondary end points	1. Change from baseline in eGFR at 6 mo 2. Proportion of participants who meet the criteria for achieving a composite renal end point at 6 mo ([*1*] a stable or improved eGFR compared with the baseline visit [≤15% reduction in eGFR], and [*2*] a ≥50% reduction in UPCR compared with the baseline) 3. Change from baseline in disease total activity score in a renal biopsy at 6 mo 4. Change from baseline in the FACIT-Fatigue score at 6 mo	1. The proportion of participants who meet the criteria for achieving a composite renal end point (a stable or improved eGFR compared with the baseline visit (≤15% reduction in eGFR), and a ≥50% reduction in UPCR compared with the baseline visit) 2. The proportion of participants with a reduction of at least 50% from baseline in UPCR 3. Change from baseline in eGFR 4. For participants with evaluable renal biopsies, the change from baseline in the activity score of the C3G histologic index score 5. The proportion of participants with evaluable renal biopsies showing decreases in C3c staining on renal biopsy from baseline 6. The proportion of participants achieving proteinuria <1 g/d 7. For participants with serum albumin levels below the lower LLN at baseline, the proportion of participants with normalization of serum albumin levels 8. For participants with serum C3 levels below the LLN at baseline, the proportion of participants with serum C3 levels above the LLN 9. The change from baseline in FACIT-Fatigue scale score

C3G, C3 glomerulopathy; FACIT-Fatigue, Functional Assessment of Chronic Illness Therapy-Fatigue; LLN, limit of normal; UPCR, urine protein:creatinine ratio.

### Data Supporting Use of Proposed End Points: Evidence and Uncertainties

#### Proteinuria

Proteinuria is a risk marker for disease progression across glomerular disorders.^11–14^ In some, such as IgA nephropathy, sufficiently large treatment effects on proteinuria are believed to predict treatment effects on disease progression.^15,16^ In patients with C3G, proteinuria is mainly associated with endocapillary hypercellularity and glomerular basement membrane double contours.^17^ These are key histopathologic features that are believed to be reflective of active C3G. Proteinuria in this context seems to be tied to disease-mediated injury to the glomerular filtration barrier (such as is seen with the membranoproliferative pattern).

As in other glomerular diseases, higher baseline levels of proteinuria in patients with C3G, *i.e*., >3.0–3.5 g/d, are associated with a higher risk of disease progression.^3,18^ Patients with persistent high-level proteinuria (exceeding 3.5 g/d) are more likely to develop kidney failure. Reductions in proteinuria over time are also associated with a lower risk of progression to kidney failure. In the retrospective Spanish Group for the Study of Glomerular Diseases (GLOSEN) cohort of 85 Spanish patients with native kidney C3G and baseline proteinuria of 3  g/d (interquartile range, 1.5–5.2), 45 patients (53%) experienced a 50% proteinuria reduction within the first 12 months from diagnosis in response to the local standard of care.^19^ The hazard ratio (HR) for progression to kidney failure (defined as an eGFR <15 ml/min per 1.73 m^2^, maintenance dialysis or preemptive kidney transplantation) in patients who experienced a 50% proteinuria reduction within the first 12 months from diagnosis was 0.96 (95% confidence interval [CI], 0.94 to 0.98) and 0.83 (95% CI, 0.69 to 0.95) at 6 and 12 months, respectively, compared with those who did not achieve such a decrease in proteinuria. In the GLOSEN cohort, there was also an inverse relationship between the slope of eGFR and the change in proteinuria over time (R=−0.33; 95% CI, −0.51 to −0.12, *P* = 0.002). No patient who showed a reduction in proteinuria over time reached kidney failure during a median follow-up of 49 months (interquartile range, 24–112).^3^ It is important to note that the outcomes in the patients enrolled in the GLOSEN cohort were not adjusted for the baseline level of proteinuria or eGFR, which may affect the relationship between proteinuria reduction and eGFR changes. Analysis of outcomes among 135 patients with biopsy-confirmed C3G in the UK Rare Kidney Disease Registry showed high lifetime risk of kidney failure^20^ and that proteinuria at the time of diagnosis was a poor predictor of kidney failure risk. However, proteinuria reduction (particularly to levels below 100 mg/mmol creatinine per day) at 12 months was associated with very substantially reduced HR 0.12 (95% CI, 0.02 to 0.62) for kidney failure over 20 years.^21^ Among patients enrolled in the C3G Registry at the University of Iowa (*n*=34 with 61 1-year follow-up spans, mean age, 22.7 years; mean eGFR, 83.1 ml/min per 1.73 m^2^; mean UPCR, 2.86 g/g; mean plasma C3, 75.1 mg/dl), the linear regression model in 34 1-year spans indicated that a 50% reduction in UPCR over 1 year is associated with a predicted 9% relative improvement in percent change from baseline in eGFR (*P* = 0.03), whereas a 30% reduction in UPCR is associated with a predicted 4.6% relative improvement in eGFR.^22^ These findings lend support to an association between proteinuria reduction and risk of progression to kidney failure and possibly stabilization or improvement in eGFR.

Proteinuria as an end point in C3G is, however, associated with uncertainties. The data supporting the use of proteinuria as an efficacy end point are limited and mainly derived from observational or retrospective studies that are prone to incomplete/incorrect diagnosis classification, data collection, and capture of outcomes. In addition, some of the reports have not been peer reviewed. An association between changes in proteinuria in response to *currently used* interventions and improvements in kidney function has not been consistently observed across studies. For example, in the report by Lomax-Browne *et al.*,^23^ among 75 patients with at least 2 years of follow-up after their diagnostic kidney biopsy, there was no significant difference in outcome-free kidney survival between those who did (*n*=37) and those who did not (*n*=38) achieve a 50% decrease in proteinuria. Moreover, while proteinuria may be associated with active C3G lesions on histopathology, just as with other glomerular diseases, irreversible sclerosing lesions can also lead to proteinuria. In such cases, proteinuria may decrease because kidney function/GFR is decreasing and may reflect an overall worsening of the disease. Hence, proteinuria must be assessed in the context of the concurrent histopathologic findings. Finally, given the available data, it is also unclear how large the treatment effect on proteinuria needs to be after 6 months to provide confidence that the treatment would reduce the risk of kidney failure over the long term. It is important to note that although proteinuria reduction is believed to be beneficial in proteinuric glomerular diseases in general, the magnitude of change that predicts benefit on the rate of loss of kidney function or progression to kidney failure may be different for each disease; for C3G, this relationship is not well understood.

#### eGFR

Patients progress through declining levels of eGFR before reaching kidney failure. As such, clinically significant treatment effects on the loss of kidney function as measured by eGFR would be expected to predict treatment effects on progression to kidney failure in patients with C3G who are at high risk of disease progression.^24,25^ There are limited data indicating that when patients with C3G are categorized on the basis of the annual rate of decline in kidney function, lower eGFR slope values over a period of follow-up time beginning at 6 months are associated with a graded reduction in the HR for progression to kidney failure.^23,26^ Analysis of outcomes in C3G in the Rare Kidney Disease Registry demonstrates a consistent association of annualized 24-month slope of eGFR with HR of kidney failure over a 20-year period. For example, the kidney failure HR for −6 ml/min per 1.73 m^2^ per year compared with stable eGFR was 1.61 (95% CI, 1.11 to 2.32).^21^ These data are limited but suggest that stabilization of eGFR or a reduction in the rate of decline of eGFR over a period of at least 6 months in patients with C3G could be associated with clinical benefit. However, uncertainties remain related to whether a 6-month period is sufficiently long enough to detect meaningful changes in kidney function. Finally, work group members recognized the potential confounding of serum creatinine-based eGFR measurements that results when drugs have reversible hemodynamic effects on eGFR or cause changes in serum creatinine levels that do not reflect changes in kidney function.

#### Histopathology

C3G is defined by the predominant deposition of complement components, and C3 deposition is believed to play a causal role in the disease.^5^ As such, for drugs designed to inhibit C3 activation, reduction in C3c staining in the kidney tissue would provide confirmation of drug activity in patients with C3G in whom C3 deposition is not expected to spontaneously decrease/resolve. What constitutes a clinically meaningful reduction in C3c staining that would correlate with improved outcomes, or whether a 6-month period is sufficient to detect a meaningful change in C3c staining intensity is unclear because of the lack of serial kidney biopsies in patients with C3G. Finally, whether currently used methods are sufficiently reliable and precise to adequately detect changes in C3c staining at month 6 in clinical trial participants is uncertain. In an animal model of C3G (complement factor H knockout mice), glomerular C3c staining was reduced within 24 hours of initiating the test intervention, namely infusion of factor H.^27^

Changes in the C3G histologic activity index may provide mechanistic evidence of a drug's efficacy and, on the basis of findings in other types of glomerular diseases (*e.g*., lupus nephritis) and experimental models of C3G, a 6-month period may be sufficient to detect changes in this index. In contrast to the lack of evidence linking the intensity of C3 staining and disease severity, biopsy activity scores appear to be directly correlated with biomarkers of systemic activation of the complement cascade.^28,29^ While the activity index was correlated with proteinuria in some patients with C3G (*i.e*., the Columbia University cohort),^1^ a similar association was not observed in others (*i.e*., the GLOSEN study).^18^ Other factors, such as the reliability of the measurement and responsiveness to change, are not well understood. The components of the activity score, including fibrinoid necrosis, are arbitrary and may change at different rates in response to successful treatment. It is also uncertain how the different histological indicators of active disease should be weighted. Finally, the adequacy of the tissue sample obtained in the biopsy and variations in processing and staining procedures may limit the ability to detect meaningful and reproducible changes in the activity score. The evidence and uncertainties regarding the three prespecified end points are summarized in Table 2.

**Table 2:** Evidence and uncertainties in C3 glomerulopathy trial end points at 6 months

PRE-Specified End Point	Evidence	Uncertainties
Proteinuria	Baseline proteinuria >3.5 g/d is associated with worse outcome^3,18^ A 50% reduction in proteinuria in the first 12 mo is associated with a lower HR for progression to kidney failure over 12 mo^19^ Inverse relationship between UPCR and eGFR slope^3^ On the basis of data from RaDaR, proteinuria reduction (particularly to levels below 100 mg/mmol creatinine per day) at 12 mo is associated with a reduced HR for kidney failure over 20 yr^20,21^ A 50% reduction in UPCR over 1 yr is associated with a predicted 9% relative improvement in percent change from baseline in eGFR in the Iowa C3G registry^22^	Inconsistent findings across studies on the effects of proteinuria reduction on progression to kidney failure, *e.g*., one study showed no difference on outcome-free kidney survival in patients with or without 50% reduction in proteinuria^23^ Randomized controlled trials are lacking to assess whether treatment effects on proteinuria predict treatment effects on loss of kidney function Relationship to irreversible structural damage is unclear Lack of data on the quantitative relationship between proteinuria reduction and progression to kidney failure
eGFR	Lower eGFR slope over a period of follow-up time beginning at 6 mo is associated with graded reduction in HR for progression to kidney failure^23,26^ Annualized 24-mo slope of eGFR associated with HR of kidney failure over a 20-yr period^20,21^	6 mo may not be an adequate duration of time over which participants need to be followed to detect meaningful treatment effects on the loss kidney function Hemodynamic or other effects of treatment on eGFR not described in studies
Histopathology	Reduced C3 deposition could indicate target engagement by therapies targeting the alternative complement pathway Disease activity index may be associated with response to treatment^1^	Disease activity index not always associated with response to treatment^18^ Unclear what alterations in the histopathology constitute a meaningful change Sampling error and lack of standardization of methods could make it challenging to detect treatment effect even if it exists Timing of histological changes not defined

C3G, C3 glomerulopathy; HR, hazard ratio; RaDaR, Rare Kidney Disease Registry; UPCR, urine protein:creatinine ratio.

### Deliberations of the Full Work Group

Members of the larger work group agreed with the characterization of the data supporting the use of the three prespecified end points. They also agreed that showing a favorable treatment effect on all three end points would provide convincing evidence of efficacy. However, the work group also noted that the interpretation and clinical significance of these end points depended in part on the population included in a trial and the mechanism of action of the drug. For example, in contrast to a nonspecific renoprotective agent, for a drug that targeted the complement pathway, reduced C3c staining and/or change in the systemic complement biomarker profiles would indicate that the drug had achieved its intended target within the kidney and systemically and lend support to the meaningfulness of observed changes in proteinuria and eGFR.

Although work group members felt that coherence among the three end points increased the likelihood that the treatment would have a favorable effect on long-term kidney outcomes, they noted it *may* be reasonable to conclude that a therapy was effective in the absence of complete coherence. For example, if favorable effects were seen on proteinuria and eGFR end points but not on the histology-based end point, this may provide sufficient evidence of clinical benefit, especially because serial biopsies in a controlled setting may not always be available, such as in pediatric patients. Members of the work group highlighted the importance of critically examining the data in its totality, including the overall consistency of findings across prespecified end points, the findings in prespecified subgroups of interest, and the size of the treatment effect. They noted that in light of the heterogeneity of C3G, a negative result would not preclude benefit of the test therapy in a subset of patients selected on the basis of more precise mechanistic criteria. The panel noted the unmet need for safe and effective treatments of post-kidney transplant recurrent C3G and of immune-complex membranoproliferative GN and recommended that further work be performed to identify end points for these conditions.

## Conclusions

C3G is a rare glomerular disease characterized by dysregulation of the alternative pathway of complement. A number of agents that target the complement system at different levels along the inflammatory cascade are being evaluated as treatments of C3G. The KHI C3G Trial Endpoint Work Group assessed the currently available data supporting the efficacy end points being used as measures of clinical benefit in two ongoing phase 3 clinical trials. The panel of key opinion leaders was also asked to opine on efficacy findings that the work group might consider to be compelling. The input of the experts was requested because of the limitations of the available data and the lack of information on the minimum thresholds for change in any of the three prespecified end points that would be considered clinically meaningful in C3G. Although there are limitations to the data supporting use of each of the three end points—proteinuria reduction, eGFR stabilization, and histopathological improvement—the C3G Trial Endpoints Work Group concluded that showing a favorable treatment effect on all three end points could provide convincing evidence of efficacy in the setting of a therapy that targeted the complement pathway. The work group also noted it might be reasonable to conclude that such a therapy was effective in the absence of complete alignment in all three end points, for example, if there was lowering of proteinuria and stabilization or improvement in eGFR with equivocal histologic outcomes. Given the limitations of the available data, the panel emphasized that its deliberations did not define degrees of change or minimum threshold for change in any of the three prespecified end points that might be considered clinically meaningful in C3G. The experts unanimously supported efforts to foster data sharing between academic and industry partners similar to work that is underway to assess the quantitative relationship between interim changes in proteinuria and kidney function outcomes in patients with FSGS (Proteinuria and GFR as Clinical Trial Endpoints in FSGS; www.is-gd.org/parasol). Such an initiative would address the gaps in current knowledge identified by the review of the end points in the aforementioned C3G trials (word count: 3410).

## Supplementary Material

## Supplemental Material

Supplemental Appendix 1. The appendix represents the more comprehensive list of references that were retrieved and reviewed by the work group members. The study includes the key citations from the supplemental appendix.
