# Supplementary material for: Developing Therapies for C3 Glomerulopathy: Report of the Kidney Health Initiative C3 Glomerulopathy Trial Endpoints Work Group
Source: Clin J Am Soc Nephrol. 2024 Jun 3;19(9):1201–8. doi: 10.2215/CJN.0000000000000505 (PMC11390019; doi:10.2215/CJN.0000000000000505)
Supplement: Supplementary file 1 [file cjasn-19-1201-s001.pdf]

ASN American Society of Nephrology · JASN Journal of the American Society of Nephrology · KIDNEY360 Accessing Our World From Every Angle · CJASN Clinical Journal of the American Society of Nephrology

ASN Journal Disclosure Form

As per ASN journal policy, I have disclosed any financial relationship or commitment held by myself and/or my spouse/partner in the past 36 months as included below. I have listed my Current Employer below to indicate there is a relationship requiring disclosure. If no relationship exists, my Current Employer is not listed.

S. Aslam reports the following:
Employer: BioCryst Pharmaceuticals, Durham, NC; Angion Biomedica, Uniondale, NY; Consultancy: Angion Biomedica, Uniondale, NY; Ownership Interest: BioCryst Pharmaceuticals; Angion Biomedica Corp, Uniondale, NY; and Patents or Royalties: Angion Biomedica Corp; Fresenius Medical Care of NA.

I understand that the information above will be published within the journal article, if accepted, and that failure to comply and/or to accurately and completely report the potential financial conflicts of interest could lead to the following: 1) Prior to publication, article rejection, or 2) Post-publication, sanctions ranging from, but not limited to, issuing a correction, reporting the inaccurate information to the authors' institution, banning authors from submitting work to ASN journals for varying lengths of time, and/or retraction of the published work.

Name: Shakil Aslam
Manuscript ID: CJASN-2024-000084R1
Manuscript Title: DEVELOPING THERAPIES FOR C3G: REPORT OF THE KIDNEY HEALTH INITIATIVE C3G TRIAL ENDPOINTS WORK GROUP
Date of Completion: April 11, 2024
Disclosure Updated Date: April 11, 2024

ASN American Society of Nephrology JASN Journal of the American Society of Nephrology KIDNEY360 Accessing Our World From Every Angle CJASN Clinical Journal of the American Society of Nephrology

ASN Journal Disclosure Form

As per ASN journal policy, I have disclosed any financial relationships or commitments I have held in the past 36 months as included below. I have listed my Current Employer below to indicate there is a relationship requiring disclosure. If no relationship exists, my Current Employer is not listed.

S. Balogun has nothing to disclose.

I understand that the information above will be published within the journal article, if accepted, and that failure to comply and/or to accurately and completely report the potential financial conflicts of interest could lead to the following: 1) Prior to publication, article rejection, or 2) Post-publication, sanctions ranging from, but not limited to, issuing a correction, reporting the inaccurate information to the authors' institution, banning authors from submitting work to ASN journals for varying lengths of time, and/or retraction of the published work.

Name: Seyi Balogun
Manuscript ID: CJASN-2024-000084R1
Manuscript Title: DEVELOPING THERAPIES FOR C3G: REPORT OF THE KIDNEY HEALTH INITIATIVE C3G TRIAL ENDPOINTS WORK GROUP
Date of Completion: May 9, 2024
Disclosure Updated Date: April 11, 2024

ASN American Society of Nephrology | JASN Journal of the American Society of Nephrology | KIDNEY360 Accessing Our World From Every Angle | CJASN Clinical Journal of the American Society of Nephrology

ASN Journal Disclosure Form

As per ASN journal policy, I have disclosed any financial relationship or commitment held by myself and/or my spouse/partner in the past 36 months as included below. I have listed my Current Employer below to indicate there is a relationship requiring disclosure. If no relationship exists, my Current Employer is not listed.

A. Bomback reports the following:
Employer: Columbia University; Consultancy: Amgen; Novartis; Kezar; Silence Therapeutics; Genentech; Visterra; Catalyst; Q32; Apellis; and Honoraria: UpToDate; Travere; Principio; Alexion; Aurinia; Calliditas; Glaxo Smith Kline; ANI.

I understand that the information above will be published within the journal article, if accepted, and that failure to comply and/or to accurately and completely report the potential financial conflicts of interest could lead to the following: 1) Prior to publication, article rejection, or 2) Post-publication, sanctions ranging from, but not limited to, issuing a correction, reporting the inaccurate information to the authors' institution, banning authors from submitting work to ASN journals for varying lengths of time, and/or retraction of the published work.

Name: Andrew S. Bomback
Manuscript ID: CJASN-2024-000084R1
Manuscript Title: DEVELOPING THERAPIES FOR C3G: REPORT OF THE KIDNEY HEALTH INITIATIVE C3G TRIAL ENDPOINTS WORK GROUP
Date of Completion: April 10, 2024
Disclosure Updated Date: May 19, 2023

ASN American Society of Nephrology | JASN Journal of the American Society of Nephrology | KIDNEY360 Accessing Our World From Every Angle | CJASN Clinical Journal of the American Society of Nephrology

ASN Journal Disclosure Form

As per ASN journal policy, I have disclosed any financial relationship or commitment held by myself and/or my spouse/partner in the past 36 months as included below. I have listed my Current Employer below to indicate there is a relationship requiring disclosure. If no relationship exists, my Current Employer is not listed.

F. Caravaca-Fontan reports the following:
Employer: Hospital Universitario 12 de Octubre, Madrid, Spain; Consultancy: Novartis, Apellis; and Research Funding: Novartis Pharmaceuticals.

I understand that the information above will be published within the journal article, if accepted, and that failure to comply and/or to accurately and completely report the potential financial conflicts of interest could lead to the following: 1) Prior to publication, article rejection, or 2) Post-publication, sanctions ranging from, but not limited to, issuing a correction, reporting the inaccurate information to the authors' institution, banning authors from submitting work to ASN journals for varying lengths of time, and/or retraction of the published work.

Name: Fernando Caravaca-Fontan
Manuscript ID: CJASN-2024-000084R1
Manuscript Title: DEVELOPING THERAPIES FOR C3G: REPORT OF THE KIDNEY HEALTH INITIATIVE C3G TRIAL ENDPOINTS WORK GROUP
Date of Completion: April 10, 2024
Disclosure Updated Date: May 19, 2023

ASN American Society of Nephrology
JASN Journal of the American Society of Nephrology
KIDNEY360 Accessing Our World From Every Angle
CJASN Clinical Journal of the American Society of Nephrology

ASN Journal Disclosure Form

As per ASN journal policy, I have disclosed any financial relationship or commitment held by myself and/or my spouse/partner in the past 36 months as included below. I have listed my Current Employer below to indicate there is a relationship requiring disclosure. If no relationship exists, my Current Employer is not listed.

H. Cook reports the following:
Employer: Imperial College London; Consultancy: Apellis Pharmaceuticals, Alexion Pharmaceuticals. Novartis, Q32 Bio; and Research Funding: Alexion Pharmaceuticals.

I understand that the information above will be published within the journal article, if accepted, and that failure to comply and/or to accurately and completely report the potential financial conflicts of interest could lead to the following: 1) Prior to publication, article rejection, or 2) Post-publication, sanctions ranging from, but not limited to, issuing a correction, reporting the inaccurate information to the authors' institution, banning authors from submitting work to ASN journals for varying lengths of time, and/or retraction of the published work.

Name: H. Terence Cook
Manuscript ID: CJASN-2024-000084R1
Manuscript Title: DEVELOPING THERAPIES FOR C3G: REPORT OF THE KIDNEY HEALTH INITIATIVE C3G TRIAL ENDPOINTS WORK GROUP
Date of Completion: April 10, 2024
Disclosure Updated Date: April 10, 2024

ASN American Society of Nephrology | JASN Journal of the American Society of Nephrology | KIDNEY360 Accessing Our World From Every Angle | CJASN Clinical Journal of the American Society of Nephrology

ASN Journal Disclosure Form

As per ASN journal policy, I have disclosed any financial relationship or commitment held by myself and/or my spouse/partner in the past 36 months as included below. I have listed my Current Employer below to indicate there is a relationship requiring disclosure. If no relationship exists, my Current Employer is not listed.

D. Decker reports the following:
Employer: Apellis Pharmaceuticals; and Ownership Interest: Apellis Pharmaceuticals.

I understand that the information above will be published within the journal article, if accepted, and that failure to comply and/or to accurately and completely report the potential financial conflicts of interest could lead to the following: 1) Prior to publication, article rejection, or 2) Post-publication, sanctions ranging from, but not limited to, issuing a correction, reporting the inaccurate information to the authors' institution, banning authors from submitting work to ASN journals for varying lengths of time, and/or retraction of the published work.

Name: Dima Decker
Manuscript ID: CJASN-2024-000084R1
Manuscript Title: DEVELOPING THERAPIES FOR C3G: REPORT OF THE KIDNEY HEALTH INITIATIVE C3G TRIAL ENDPOINTS WORK GROUP
Date of Completion: April 10, 2024
Disclosure Updated Date: April 10, 2024

ASN
American Society of Nephrology
JASN
Journal of the American Society of Nephrology
KIDNEY360
Accessing Our World From Every Angle
CJASN
Clinical Journal of the American Society of Nephrology

ASN Journal Disclosure Form

As per ASN journal policy, I have disclosed any financial relationship or commitment held by myself and/or my spouse/partner in the past 36 months as included below. I have listed my Current Employer below to indicate there is a relationship requiring disclosure. If no relationship exists, my Current Employer is not listed.

D. Feldman has nothing to disclose.

I understand that the information above will be published within the journal article, if accepted, and that failure to comply and/or to accurately and completely report the potential financial conflicts of interest could lead to the following: 1) Prior to publication, article rejection, or 2) Post-publication, sanctions ranging from, but not limited to, issuing a correction, reporting the inaccurate information to the authors' institution, banning authors from submitting work to ASN journals for varying lengths of time, and/or retraction of the published work.

Name: David L. Feldman
Manuscript ID: CJASN-2024-000084R1
Manuscript Title: DEVELOPING THERAPIES FOR C3G: REPORT OF THE KIDNEY HEALTH INITIATIVE C3G TRIAL ENDPOINTS WORK GROUP
Date of Completion: April 9, 2024
Disclosure Updated Date: April 9, 2024

ASN American Society of Nephrology | JASN Journal of the American Society of Nephrology | KIDNEY360 Accessing Our World From Every Angle | CJASN Clinical Journal of the American Society of Nephrology

ASN Journal Disclosure Form

As per ASN journal policy, I have disclosed any financial relationships or commitments I have held in the past 36 months as included below. I have listed my Current Employer below to indicate there is a relationship requiring disclosure. If no relationship exists, my Current Employer is not listed.

V. Fremeaux Bacchi reports the following:
Consultancy: Served as consultant for Alexion Pharmaceuticals, Apellis, Sobi, UCB, BioCryps, Novartis and Roche;; Research Funding: Alexion Pharmaceuticals; Honoraria: Alexion Pharmaceuticals, Apellis, Sobi, UCB, BioCryps, Novartis and Roche; and Advisory or Leadership Role: Alexion Pharmaceuticals, Apellis, Sobi, BioCryps, Novartis.

I understand that the information above will be published within the journal article, if accepted, and that failure to comply and/or to accurately and completely report the potential financial conflicts of interest could lead to the following: 1) Prior to publication, article rejection, or 2) Post-publication, sanctions ranging from, but not limited to, issuing a correction, reporting the inaccurate information to the authors' institution, banning authors from submitting work to ASN journals for varying lengths of time, and/or retraction of the published work.

Name: Veronique Fremeaux Bacchi
Manuscript ID: CJASN-2024-000084R2
Manuscript Title: DEVELOPING THERAPIES FOR C3G: REPORT OF THE KIDNEY HEALTH INITIATIVE C3G TRIAL ENDPOINTS WORK GROUP
Date of Completion: May 14, 2024
Disclosure Updated Date: May 14, 2024

ASN American Society of Nephrology | JASN Journal of the American Society of Nephrology | KIDNEY360 Accessing Our World From Every Angle | CJASN Clinical Journal of the American Society of Nephrology

ASN Journal Disclosure Form

As per ASN journal policy, I have disclosed any financial relationship or commitment held by myself and/or my spouse/partner in the past 36 months as included below. I have listed my Current Employer below to indicate there is a relationship requiring disclosure. If no relationship exists, my Current Employer is not listed.

D. Gale reports the following:
Employer: University College London; Consultancy: Novartis; Alexion; Calliditas; Britannia; Vifor; Judo Bio; Sofinnova; Adnovate; Sanofi; Alnylam; Research Funding: Travere; Sanofi; Pfizer; Novartis; and Other Interests or Relationships: Trustee for AlportUK; Chair of UK Kidney Association Rare Diseases Committee.

I understand that the information above will be published within the journal article, if accepted, and that failure to comply and/or to accurately and completely report the potential financial conflicts of interest could lead to the following: 1) Prior to publication, article rejection, or 2) Post-publication, sanctions ranging from, but not limited to, issuing a correction, reporting the inaccurate information to the authors' institution, banning authors from submitting work to ASN journals for varying lengths of time, and/or retraction of the published work.

Name: Daniel P. Gale
Manuscript ID: CJASN-2024-000084R1
Manuscript Title: DEVELOPING THERAPIES FOR C3G: REPORT OF THE KIDNEY HEALTH INITIATIVE C3G TRIAL ENDPOINTS WORK GROUP
Date of Completion: April 15, 2024
Disclosure Updated Date: March 27, 2024

ASN American Society of Nephrology · JASN Journal of the American Society of Nephrology · KIDNEY360 Accessing Our World From Every Angle · CJASN Clinical Journal of the American Society of Nephrology

ASN Journal Disclosure Form

As per ASN journal policy, I have disclosed any financial relationship or commitment held by myself and/or my spouse/partner in the past 36 months as included below. I have listed my Current Employer below to indicate there is a relationship requiring disclosure. If no relationship exists, my Current Employer is not listed.

A. Gooch reports the following:
Employer: BioCryst Pharmaceuticals; and Ownership Interest: vTv Therapeutics; BioCryst Pharmaceuticals.

I understand that the information above will be published within the journal article, if accepted, and that failure to comply and/or to accurately and completely report the potential financial conflicts of interest could lead to the following: 1) Prior to publication, article rejection, or 2) Post-publication, sanctions ranging from, but not limited to, issuing a correction, reporting the inaccurate information to the authors' institution, banning authors from submitting work to ASN journals for varying lengths of time, and/or retraction of the published work.

Name: Ann Gooch
Manuscript ID: CJASN-2024-000084R1
Manuscript Title: DEVELOPING THERAPIES FOR C3G: REPORT OF THE KIDNEY HEALTH INITIATIVE C3G TRIAL ENDPOINTS WORK GROUP
Date of Completion: April 9, 2024
Disclosure Updated Date: April 9, 2024

ASN
American Society of Nephrology
JASN
Journal of the American Society of Nephrology
KIDNEY360
Accessing Our World From Every Angle
CJASN
Clinical Journal of the American Society of Nephrology

ASN Journal Disclosure Form

As per ASN journal policy, I have disclosed any financial relationship or commitment held by myself and/or my spouse/partner in the past 36 months as included below. I have listed my Current Employer below to indicate there is a relationship requiring disclosure. If no relationship exists, my Current Employer is not listed.

S. Johnson reports the following:
Employer: Newcastle Upon Tyne Hospitals NHS Foundation Trust;; Honoraria: Alexion - honoraria for talks and advisory boards. Paid to my host institution; Novartis - honoraria for talks and advisory boards. Paid to my host institution; Advisory or Leadership Role: Scientific Advisory Board for aHUS Global Registry sponsored by Alexion Pharmaceuticals. Payment was made directly from Alexion to my employer. Finished role in November 2021.; Research Secretary for British Association for Paediatric NEphrology until May 2023 - unpaid; Co-chair BAPN Clinical Studies Group - unpaid, ongoing; Chair, STEC HUS Rare Disease Group, UK Kidney Association - unpaid, ongoing; and Other Interests or Relationships: Member of grant committee for kidney research UK; Trustee of Northern COunties Kidney Research Fund charity.

I understand that the information above will be published within the journal article, if accepted, and that failure to comply and/or to accurately and completely report the potential financial conflicts of interest could lead to the following: 1) Prior to publication, article rejection, or 2) Post-publication, sanctions ranging from, but not limited to, issuing a correction, reporting the inaccurate information to the authors' institution, banning authors from submitting work to ASN journals for varying lengths of time, and/or retraction of the published work.

Name: Sally A. Johnson
Manuscript ID: CJASN-2024-000084R1
Manuscript Title: DEVELOPING THERAPIES FOR C3G: REPORT OF THE KIDNEY HEALTH INITIATIVE C3G TRIAL ENDPOINTS WORK GROUP
Date of Completion: April 10, 2024
Disclosure Updated Date: April 10, 2024

ASN American Society of Nephrology · JASN Journal of the American Society of Nephrology · KIDNEY360 Accessing Our World From Every Angle · CJASN Clinical Journal of the American Society of Nephrology

ASN Journal Disclosure Form

As per ASN journal policy, I have disclosed any financial relationship or commitment held by myself and/or my spouse/partner in the past 36 months as included below. I have listed my Current Employer below to indicate there is a relationship requiring disclosure. If no relationship exists, my Current Employer is not listed.

C. Licht reports the following:
Employer: The Hospital for Sick Children; Consultancy: Alexion, AstraZeneca Rare Disease; Apellis Pharmaceuticals, Inc.; Novartis, Pfizer Inc.; Sobi; Honoraria: Alexion, AstraZeneca Rare Disease; Apellis Pharmaceuticals, Inc.; Novartis, Pfizer Inc.; Sobi; Patents or Royalties: [International #: WO 2007/038995 A1]; [U.S. #: 11/992,194]; [CSL Behring #: 2005_M006_A105]; [Finnegan #: 06478.1518-00000]; Title: Factor H for the treatment of chronic nephropathies and production thereof.; Advisory or Leadership Role: Editorial Boards: Kidney International; Nephrology Dialysis Transplantation; Pediatric Nephrology; Advisory Committees: Alexion, AstraZeneca Rare Disease (SAB, Global aHUS Registry); DOUBLE PRO-TECT Alport (DSMB); Catalyst Biosciences?; Argenx ? Axio Research (DSMB); OPKO Health, Inc. (DSMB, CTAP101-CL-3007); and Speakers Bureau: Alexion, AstraZeneca Rare Disease; Novartis.

I understand that the information above will be published within the journal article, if accepted, and that failure to comply and/or to accurately and completely report the potential financial conflicts of interest could lead to the following: 1) Prior to publication, article rejection, or 2) Post-publication, sanctions ranging from, but not limited to, issuing a correction, reporting the inaccurate information to the authors' institution, banning authors from submitting work to ASN journals for varying lengths of time, and/or retraction of the published work.

Name: Christoph Licht
Manuscript ID: CJASN-2024-000084R1
Manuscript Title: DEVELOPING THERAPIES FOR C3G: REPORT OF THE KIDNEY HEALTH INITIATIVE C3G TRIAL ENDPOINTS WORK GROUP.
Date of Completion: April 8, 2024
Disclosure Updated Date: April 8, 2024

ASN
American Society of Nephrology
JASN
Journal of the American Society of Nephrology
KIDNEY360
Accessing Our World From Every Angle
CJASN
Clinical Journal of the American Society of Nephrology

ASN Journal Disclosure Form

As per ASN journal policy, I have disclosed any financial relationships or commitments I have held in the past 36 months as included below. I have listed my Current Employer below to indicate there is a relationship requiring disclosure. If no relationship exists, my Current Employer is not listed.

M. Lim reports the following:
Employer: American Society of Nephrology; and Advisory or Leadership Role: Voting Member, Scientific Advisory Board, Quebec Consortium for Drug Discovery (CQDM); Roster of Experts in Digital Health, World Health Organization; Scientific Advisory Committee, California Healthy Nail Salon Collaborative.

I understand that the information above will be published within the journal article, if accepted, and that failure to comply and/or to accurately and completely report the potential financial conflicts of interest could lead to the following: 1) Prior to publication, article rejection, or 2) Post-publication, sanctions ranging from, but not limited to, issuing a correction, reporting the inaccurate information to the authors' institution, banning authors from submitting work to ASN journals for varying lengths of time, and/or retraction of the published work.

Name: Mark D. Lim
Manuscript ID: CJASN-2024-000084R1
Manuscript Title: DEVELOPING THERAPIES FOR C3G: REPORT OF THE KIDNEY HEALTH INITIATIVE C3G TRIAL ENDPOINTS WORK GROUP
Date of Completion: May 7, 2024
Disclosure Updated Date: June 14, 2023

ASN American Society of Nephrology
JASN Journal of the American Society of Nephrology
KIDNEY360 Accessing Our World From Every Angle
CJASN Clinical Journal of the American Society of Nephrology

ASN Journal Disclosure Form

As per ASN journal policy, I have disclosed any financial relationship or commitment held by myself and/or my spouse/partner in the past 36 months as included below. I have listed my Current Employer below to indicate there is a relationship requiring disclosure. If no relationship exists, my Current Employer is not listed.

M. Mathur reports the following:
Employer: Visterra Inc

I understand that the information above will be published within the journal article, if accepted, and that failure to comply and/or to accurately and completely report the potential financial conflicts of interest could lead to the following: 1) Prior to publication, article rejection, or 2) Post-publication, sanctions ranging from, but not limited to, issuing a correction, reporting the inaccurate information to the authors' institution, banning authors from submitting work to ASN journals for varying lengths of time, and/or retraction of the published work.

Name: Mohit Mathur
Manuscript ID: CJASN-2024-000084R1
Manuscript Title: DEVELOPING THERAPIES FOR C3G: REPORT OF THE KIDNEY HEALTH INITIATIVE C3G TRIAL ENDPOINTS WORK GROUP
Date of Completion: April 11, 2024
Disclosure Updated Date: August 31, 2023

ASN American Society of Nephrology | JASN Journal of the American Society of Nephrology | KIDNEY360 Accessing Our World From Every Angle | CJASN Clinical Journal of the American Society of Nephrology

ASN Journal Disclosure Form

As per ASN journal policy, I have disclosed any financial relationships or commitments I have held in the past 36 months as included below. I have listed my Current Employer below to indicate there is a relationship requiring disclosure. If no relationship exists, my Current Employer is not listed.

M. Meier reports the following:
Employer: Novartis Pharma; and Ownership Interest: Novartis Pharma.

I understand that the information above will be published within the journal article, if accepted, and that failure to comply and/or to accurately and completely report the potential financial conflicts of interest could lead to the following: 1) Prior to publication, article rejection, or 2) Post-publication, sanctions ranging from, but not limited to, issuing a correction, reporting the inaccurate information to the authors' institution, banning authors from submitting work to ASN journals for varying lengths of time, and/or retraction of the published work.

Name: Matthias Meier
Manuscript ID: Disclosure Form for CJASN-2024-000084R2
Manuscript Title: DEVELOPING THERAPIES FOR C3G: REPORT OF THE KIDNEY HEALTH INITIATIVE C3G TRIAL ENDPOINTS WORK GROUP
Date of Completion: May 23, 2024
Disclosure Updated Date: May 23, 2024

ASN
American Society of Nephrology
JASN
Journal of the American Society of Nephrology
KIDNEY360
Accessing Our World From Every Angle
CJASN
Clinical Journal of the American Society of Nephrology

ASN Journal Disclosure Form

As per ASN journal policy, I have disclosed any financial relationships or commitments I have held in the past 36 months as included below. I have listed my Current Employer below to indicate there is a relationship requiring disclosure. If no relationship exists, my Current Employer is not listed.

K. Mistry has nothing to disclose.

I understand that the information above will be published within the journal article, if accepted, and that failure to comply and/or to accurately and completely report the potential financial conflicts of interest could lead to the following: 1) Prior to publication, article rejection, or 2) Post-publication, sanctions ranging from, but not limited to, issuing a correction, reporting the inaccurate information to the authors' institution, banning authors from submitting work to ASN journals for varying lengths of time, and/or retraction of the published work.

Name: Kirtida Mistry
Manuscript ID: CJASN-2024-000084
Manuscript Title: DEVELOPING THERAPIES FOR C3G: REPORT OF THE KIDNEY HEALTH INITIATIVE C3G TRIAL ENDPOINTS WORK GROUP
Date of Completion: May 6, 2024
Disclosure Updated Date: May 6, 2024

ASN American Society of Nephrology
JASN Journal of the American Society of Nephrology
KIDNEY360 Accessing Our World From Every Angle
CJASN Clinical Journal of the American Society of Nephrology

ASN Journal Disclosure Form

As per ASN journal policy, I have disclosed any financial relationships or commitments I have held in the past 36 months as included below. I have listed my Current Employer below to indicate there is a relationship requiring disclosure. If no relationship exists, my Current Employer is not listed.

C. Nester reports the following:
Employer: University of Iowa; Consultancy: Advisory Board -Biocryst, Novartis, Apellis, Alexion, Kira, Silence Therapeutics; Research Funding: Retrophin - Pediatric Recruiting Site for the Duet Trial; Site PI - C3G Trial; Novartis - Site PI - C3G Trial ;Apellis - Site PI - C3G Trial, Biocryst; and Patents or Royalties: UpToDate - TMA Syndromes.

I understand that the information above will be published within the journal article, if accepted, and that failure to comply and/or to accurately and completely report the potential financial conflicts of interest could lead to the following: 1) Prior to publication, article rejection, or 2) Post-publication, sanctions ranging from, but not limited to, issuing a correction, reporting the inaccurate information to the authors' institution, banning authors from submitting work to ASN journals for varying lengths of time, and/or retraction of the published work.

Name: Carla M Nester
Manuscript ID: CJASN Manuscript CJASN-2024-000084R1
Manuscript Title: DEVELOPING THERAPIES FOR C3G: REPORT OF THE KIDNEY HEALTH INITIATIVE C3G TRIAL ENDPOINTS WORK GROUP
Date of Completion: May 9, 2024
Disclosure Updated Date: March 5, 2024

ASN
American Society of Nephrology
JASN
Journal of the American Society of Nephrology
KIDNEY360
Accessing Our World From Every Angle
CJASN
Clinical Journal of the American Society of Nephrology

ASN Journal Disclosure Form

As per ASN journal policy, I have disclosed any financial relationships or commitments I have held in the past 36 months as included below. I have listed my Current Employer below to indicate there is a relationship requiring disclosure. If no relationship exists, my Current Employer is not listed.

M. Pickering reports the following:
Employer: Imperial College, London; Consultancy: I have provided clinical and scientific advisory board roles for Alexion, Annexon, Biocryst, Complement Therapeutics, Gyroscope, PureSpring, Sobi.; Ownership Interest: My wife works for Vertex Pharma and hold stock in that company.; Research Funding: I receive research funding from Omeros.; and Honoraria: I have received honoraria for clinical and scientific advisory board roles for Alexion, Annexon, Biocryst, Complement Therapeutics, Gyroscope, PureSpring, Sobi.

I understand that the information above will be published within the journal article, if accepted, and that failure to comply and/or to accurately and completely report the potential financial conflicts of interest could lead to the following: 1) Prior to publication, article rejection, or 2) Post-publication, sanctions ranging from, but not limited to, issuing a correction, reporting the inaccurate information to the authors' institution, banning authors from submitting work to ASN journals for varying lengths of time, and/or retraction of the published work.

Name: Matthew C. Pickering
Manuscript ID: CJASN-2024-000084
Manuscript Title: DEVELOPING THERAPIES FOR C3G: REPORT OF THE KIDNEY HEALTH INITIATIVE C3G TRIAL ENDPOINTS WORK GROUP
Date of Completion: May 7, 2024
Disclosure Updated Date: March 20, 2024

ASN Journal Disclosure Form

As per ASN journal policy, I have disclosed any financial relationship or commitment held by myself and/or my spouse/partner in the past 36 months as included below. I have listed my Current Employer below to indicate there is a relationship requiring disclosure. If no relationship exists, my Current Employer is not listed.

C. Portillo reports the following:
Employer: American Society of Nephrology

I understand that the information above will be published within the journal article, if accepted, and that failure to comply and/or to accurately and completely report the potential financial conflicts of interest could lead to the following: 1) Prior to publication, article rejection, or 2) Post-publication, sanctions ranging from, but not limited to, issuing a correction, reporting the inaccurate information to the authors' institution, banning authors from submitting work to ASN journals for varying lengths of time, and/or retraction of the published work.

Name: Cesia Portillo
Manuscript ID: CJASN-2024-000084R1
Manuscript Title: DEVELOPING THERAPIES FOR C3G: REPORT OF THE KIDNEY HEALTH INITIATIVE C3G TRIAL ENDPOINTS WORK GROUP
Date of Completion: April 10, 2024
Disclosure Updated Date: April 10, 2024

ASN American Society of Nephrology | JASN Journal of the American Society of Nephrology | KIDNEY360 Accessing Our World From Every Angle | CJASN Clinical Journal of the American Society of Nephrology

ASN Journal Disclosure Form

As per ASN journal policy, I have disclosed any financial relationships or commitments I have held in the past 36 months as included below. I have listed my Current Employer below to indicate there is a relationship requiring disclosure. If no relationship exists, my Current Employer is not listed.

M. Praga reports the following:
Employer: Complutense University, Madrid, Spain; Consultancy: Apellis, Alexion, Astra Zeneca, Travere, Silence, Novartis, GSK, Otsuka, Vifor, Stada; Honoraria: Alexion, Travere, Novartis, GSK, Otsuka, Vifor, Stada; and Patents or Royalties: UpToDate.

I understand that the information above will be published within the journal article, if accepted, and that failure to comply and/or to accurately and completely report the potential financial conflicts of interest could lead to the following: 1) Prior to publication, article rejection, or 2) Post-publication, sanctions ranging from, but not limited to, issuing a correction, reporting the inaccurate information to the authors' institution, banning authors from submitting work to ASN journals for varying lengths of time, and/or retraction of the published work.

Name: Manuel Praga
Manuscript ID: CJASN-2024-000084R1
Manuscript Title: DEVELOPING THERAPIES FOR C3G: REPORT OF THE KIDNEY HEALTH INITIATIVE C3G TRIAL ENDPOINTS WORK GROUP
Date of Completion: May 7, 2024
Disclosure Updated Date: May 7, 2024

ASN American Society of Nephrology
JASN Journal of the American Society of Nephrology
KIDNEY360 Accessing Our World From Every Angle
CJASN Clinical Journal of the American Society of Nephrology

ASN Journal Disclosure Form

As per ASN journal policy, I have disclosed any financial relationships or commitments I have held in the past 36 months as included below. I have listed my Current Employer below to indicate there is a relationship requiring disclosure. If no relationship exists, my Current Employer is not listed.

G. Remuzzi reports the following:
Consultancy: Consulting fees: Silence Therapeutics, Otsuka, AstraZeneca Pharmaceuticals, Alexion Pharmaceuticals.; Honoraria: Novartis Pharma AG; and Advisory or Leadership Role: Giuseppe Remuzzi is member of numerous Editorial Boards of Scientific Medical Journals.

I understand that the information above will be published within the journal article, if accepted, and that failure to comply and/or to accurately and completely report the potential financial conflicts of interest could lead to the following: 1) Prior to publication, article rejection, or 2) Post-publication, sanctions ranging from, but not limited to, issuing a correction, reporting the inaccurate information to the authors' institution, banning authors from submitting work to ASN journals for varying lengths of time, and/or retraction of the published work.

Name: Giuseppe Remuzzi
Manuscript ID: CJASN-2024-000084R1
Manuscript Title: DEVELOPING THERAPIES FOR C3G: REPORT OF THE KIDNEY HEALTH INITIATIVE C3G TRIAL ENDPOINTS WORK GROUP
Date of Completion: May 10, 2024
Disclosure Updated Date: May 7, 2024

ASN American Society of Nephrology | JASN Journal of the American Society of Nephrology | KIDNEY360 Accessing Our World From Every Angle | CJASN Clinical Journal of the American Society of Nephrology

ASN Journal Disclosure Form

As per ASN journal policy, I have disclosed any financial relationship or commitment held by myself and/or my spouse/partner in the past 36 months as included below. I have listed my Current Employer below to indicate there is a relationship requiring disclosure. If no relationship exists, my Current Employer is not listed.

V. Selvarajah reports the following:
Employer: AstraZeneca; and Ownership Interest: I am an AstraZeneca stockholder.

I understand that the information above will be published within the journal article, if accepted, and that failure to comply and/or to accurately and completely report the potential financial conflicts of interest could lead to the following: 1) Prior to publication, article rejection, or 2) Post-publication, sanctions ranging from, but not limited to, issuing a correction, reporting the inaccurate information to the authors' institution, banning authors from submitting work to ASN journals for varying lengths of time, and/or retraction of the published work.

Name: Viknesh Selvarajah
Manuscript ID: CJASN-2024-000084R1
Manuscript Title: DEVELOPING THERAPIES FOR C3G: REPORT OF THE KIDNEY HEALTH INITIATIVE C3G TRIAL ENDPOINTS WORK GROUP
Date of Completion: April 9, 2024
Disclosure Updated Date: April 9, 2024

ASN American Society of Nephrology | JASN Journal of the American Society of Nephrology | KIDNEY360 Accessing Our World From Every Angle | CJASN Clinical Journal of the American Society of Nephrology

ASN Journal Disclosure Form

As per ASN journal policy, I have disclosed any financial relationship or commitment held by myself and/or my spouse/partner in the past 36 months as included below. I have listed my Current Employer below to indicate there is a relationship requiring disclosure. If no relationship exists, my Current Employer is not listed.

R. Smith reports the following:
Employer: University of Iowa; Consultancy: Novartis; Research Funding: The Binding Site; Honoraria: I have received honoraria for giving some academic talks.; Advisory or Leadership Role: Several academic journals; and Other Interests or Relationships: Secretary for Kidneeds, a not-for-profit fund of the Greater Cedar Rapids Community Foundation dedicated to the study of Dense Deposit Disease and C3 Glomerulopathy.

I understand that the information above will be published within the journal article, if accepted, and that failure to comply and/or to accurately and completely report the potential financial conflicts of interest could lead to the following: 1) Prior to publication, article rejection, or 2) Post-publication, sanctions ranging from, but not limited to, issuing a correction, reporting the inaccurate information to the authors' institution, banning authors from submitting work to ASN journals for varying lengths of time, and/or retraction of the published work.

Name: Richard J. Smith
Manuscript ID: Disclosure Form for CJASN-2024-000084R1
Manuscript Title: DEVELOPING THERAPIES FOR C3G: REPORT OF THE KIDNEY HEALTH INITIATIVE C3G TRIAL ENDPOINTS WORK GROUP
Date of Completion: April 10, 2024
Disclosure Updated Date: April 1, 2024

ASN
American Society of Nephrology
JASN
Journal of the American Society of Nephrology
KIDNEY360
Accessing Our World From Every Angle
CJASN
Clinical Journal of the American Society of Nephrology

ASN Journal Disclosure Form

As per ASN journal policy, I have disclosed any financial relationship or commitment held by myself and/or my spouse/partner in the past 36 months as included below. I have listed my Current Employer below to indicate there is a relationship requiring disclosure. If no relationship exists, my Current Employer is not listed.

H. Tabriziani reports the following:
Employer: Natera; Consultancy: Natera; Ownership Interest: Natera; Patents or Royalties: Natera; Advisory or Leadership Role: Natera; Speakers Bureau: Natera; and Other Interests or Relationships: HossMed, Inc.

I understand that the information above will be published within the journal article, if accepted, and that failure to comply and/or to accurately and completely report the potential financial conflicts of interest could lead to the following: 1) Prior to publication, article rejection, or 2) Post-publication, sanctions ranging from, but not limited to, issuing a correction, reporting the inaccurate information to the authors' institution, banning authors from submitting work to ASN journals for varying lengths of time, and/or retraction of the published work.

Name: Hossein Tabriziani
Manuscript ID: CJASN-2024-000084R1
Manuscript Title: DEVELOPING THERAPIES FOR C3G: REPORT OF THE KIDNEY HEALTH INITIATIVE C3G TRIAL ENDPOINTS WORK GROUP
Date of Completion: April 11, 2024
Disclosure Updated Date: September 18, 2023

ASN
American Society of Nephrology
JASN
Journal of the American Society of Nephrology
KIDNEY360
Accessing Our World From Every Angle
CJASN
Clinical Journal of the American Society of Nephrology

ASN Journal Disclosure Form

As per ASN journal policy, I have disclosed any financial relationship or commitment held by myself and/or my spouse/partner in the past 36 months as included below. I have listed my Current Employer below to indicate there is a relationship requiring disclosure. If no relationship exists, my Current Employer is not listed.

A. Thompson reports the following:
Employer: Food and Drug Administration; Consultancy: My husband does consulting related to commercial litigation; however, this work would not be construed as resulting in an actual, potential, or perceived conflict related to my work.; and Ownership Interest: We hold stock, however none of our investments might be construed as resulting in an actual, potential, or perceived conflict related to my work. Let me know if you need additional information.

I understand that the information above will be published within the journal article, if accepted, and that failure to comply and/or to accurately and completely report the potential financial conflicts of interest could lead to the following: 1) Prior to publication, article rejection, or 2) Post-publication, sanctions ranging from, but not limited to, issuing a correction, reporting the inaccurate information to the authors' institution, banning authors from submitting work to ASN journals for varying lengths of time, and/or retraction of the published work.

Name: Aliza M. Thompson
Manuscript ID: CJASN-2024-000084R1
Manuscript Title: DEVELOPING THERAPIES FOR C3G: REPORT OF THE KIDNEY HEALTH INITIATIVE C3G TRIAL ENDPOINTS WORK GROUP
Date of Completion: April 9, 2024
Disclosure Updated Date: April 9, 2024

ASN American Society of Nephrology | JASN Journal of the American Society of Nephrology | KIDNEY360 Accessing Our World From Every Angle | CJASN Clinical Journal of the American Society of Nephrology

ASN Journal Disclosure Form

As per ASN journal policy, I have disclosed any financial relationship or commitment held by myself and/or my spouse/partner in the past 36 months as included below. I have listed my Current Employer below to indicate there is a relationship requiring disclosure. If no relationship exists, my Current Employer is not listed.

H. Trachtman reports the following:
Employer: University of Michigan Adjunct Professor of Pediatrics; RenalStrategies LLC; Consultancy: Otsuka (DSMB Chair), Bristol Meyers Squibb (inactive), Chemocentryx (DMSB), Goldfinch Bio (inactive), Travere Therapeutics, Natera (RenaSight) (inactive), Angion (inactive), Akebia (inactive), Walden, Aclipse, Boehringer-Ingelheim, PhaseV, Maze Therapeutics, Alexion/Astra Zeneca, Eloxx Pharmaceuticals, Dimerix, ProKidney, NephCure Kidney International, Kaneka (inactive), Astellas (inactive), Complexa (inactive); Ownership Interest: Aclipse; PhaseV; Honoraria: Attendance at glomerular disease panels organized by Reata and Astellas, Advisory Board for Otsuka, Travere Therapeutics; and Advisory or Leadership Role: DSMB RIVUR Trial (completed) ; DSMB bumetanide-seizure trial (completed); Chair, DSMB Otsuka trials; DSMB, ANCA vasculitis, Chemocentryx (completed); Steering Committee, Abatacept Trial, BMS (completed); DUPRO Steering Committee, Travere Therapeutics; Steering Committee, Goldfinch Bio; ProKidney, Scientific Advisory Board); MEDCAC committee member; KHI Board of Directors; Editorial Board, Pediatric Nephrology, Kidney360, and Glomerular Diseases; Editor, Expert Opinion on Emerging Drugs.

I understand that the information above will be published within the journal article, if accepted, and that failure to comply and/or to accurately and completely report the potential financial conflicts of interest could lead to the following: 1) Prior to publication, article rejection, or 2) Post-publication, sanctions ranging from, but not limited to, issuing a correction, reporting the inaccurate information to the authors' institution, banning authors from submitting work to ASN journals for varying lengths of time, and/or retraction of the published work.

Name: Howard Trachtman
Manuscript ID: CJASN-2024-000084R1
Manuscript Title: DEVELOPING THERAPIES FOR C3G: REPORT OF THE KIDNEY HEALTH INITIATIVE C3G TRIAL ENDPOINTS WORK GROUP
Date of Completion: April 10, 2024
Disclosure Updated Date: April 10, 2024

ASN American Society of Nephrology | JASN Journal of the American Society of Nephrology | KIDNEY360 Accessing Our World From Every Angle | CJASN Clinical Journal of the American Society of Nephrology

ASN Journal Disclosure Form

As per ASN journal policy, I have disclosed any financial relationships or commitments I have held in the past 36 months as included below. I have listed my Current Employer below to indicate there is a relationship requiring disclosure. If no relationship exists, my Current Employer is not listed.

N. Van De Kar reports the following:
Employer: Radboudumc, Nijmegen, the Netherlands; Consultancy: Roche , Novartis, and Alexion consultancy fees; and Speakers Bureau: novartis.

I understand that the information above will be published within the journal article, if accepted, and that failure to comply and/or to accurately and completely report the potential financial conflicts of interest could lead to the following: 1) Prior to publication, article rejection, or 2) Post-publication, sanctions ranging from, but not limited to, issuing a correction, reporting the inaccurate information to the authors' institution, banning authors from submitting work to ASN journals for varying lengths of time, and/or retraction of the published work.

Name: Nicole Van De Kar
Manuscript ID: CJASN-2024-000084R1
Manuscript Title: DEVELOPING THERAPIES FOR C3G: REPORT OF THE KIDNEY HEALTH INITIATIVE C3G TRIAL ENDPOINTS WORK GROUP
Date of Completion: May 6, 2024
Disclosure Updated Date: May 6, 2024

ASN American Society of Nephrology
JASN Journal of the American Society of Nephrology
KIDNEY360 Accessing Our World From Every Angle
CJASN Clinical Journal of the American Society of Nephrology

ASN Journal Disclosure Form

As per ASN journal policy, I have disclosed any financial relationship or commitment held by myself and/or my spouse/partner in the past 36 months as included below. I have listed my Current Employer below to indicate there is a relationship requiring disclosure. If no relationship exists, my Current Employer is not listed.

Y. Wang reports the following:
Employer: Novartis Pharmaceuticals Corporation; and Ownership Interest: Novartis Pharmaceuticals Corporation.

I understand that the information above will be published within the journal article, if accepted, and that failure to comply and/or to accurately and completely report the potential financial conflicts of interest could lead to the following: 1) Prior to publication, article rejection, or 2) Post-publication, sanctions ranging from, but not limited to, issuing a correction, reporting the inaccurate information to the authors' institution, banning authors from submitting work to ASN journals for varying lengths of time, and/or retraction of the published work.

Name: Yaqin Wang
Manuscript ID: CJASN-2024-000084R1
Manuscript Title: DEVELOPING THERAPIES FOR C3G: REPORT OF THE KIDNEY HEALTH INITIATIVE C3G TRIAL ENDPOINTS WORK GROUP
Date of Completion: April 9, 2024
Disclosure Updated Date: April 9, 2024

ASN American Society of Nephrology JASN Journal of the American Society of Nephrology KIDNEY360 Accessing Our World From Every Angle CJASN Clinical Journal of the American Society of Nephrology

ASN Journal Disclosure Form

As per ASN journal policy, I have disclosed any financial relationships or commitments I have held in the past 36 months as included below. I have listed my Current Employer below to indicate there is a relationship requiring disclosure. If no relationship exists, my Current Employer is not listed.

E. Wong reports the following:
Employer: Newcastle upon Tyne Hospitals NHS Foundation Trust; Consultancy: Novartis, Apellis, Biocryst, Arrowhead; Honoraria: Alexion; Novartis; Advisory or Leadership Role: Novartis; Biocryst, Apellis; Speakers Bureau: Alexion; Novartis; and Other Interests or Relationships: Chair of MPGN, DDD and C3G Rare Disease Group as part of UKKA RaDaR.

I understand that the information above will be published within the journal article, if accepted, and that failure to comply and/or to accurately and completely report the potential financial conflicts of interest could lead to the following: 1) Prior to publication, article rejection, or 2) Post-publication, sanctions ranging from, but not limited to, issuing a correction, reporting the inaccurate information to the authors' institution, banning authors from submitting work to ASN journals for varying lengths of time, and/or retraction of the published work.

Name: Edwin Kwan Soon Wong
Manuscript ID: CJASN-2024-000084
Manuscript Title: DEVELOPING THERAPIES FOR C3G: REPORT OF THE KIDNEY HEALTH INITIATIVE C3G TRIAL ENDPOINTS WORK GROUP
Date of Completion: May 14, 2024
Disclosure Updated Date: May 14, 2024
