# Supplementary material for: Developing Therapies for C3 Glomerulopathy: Report of the Kidney Health Initiative C3 Glomerulopathy Trial Endpoints Work Group
Source: Clin J Am Soc Nephrol. 2024 Jun 3;19(9):1201–8. doi: 10.2215/CJN.0000000000000505 (PMC11390019; doi:10.2215/CJN.0000000000000505)
Supplement: Supplementary file 2 [file cjasn-19-1201-s002.pdf]

Supplemental File

Full Reference List (in alphabetical order)

Alchi B, Jayne D. Membranoproliferative glomerulonephritis. *Pediatr Nephrol*. 2010;25(8):1409-18. doi: 10.1007/s00467-009-1322-7

Appel GB. Mycophenolate Mofetil in Combination with Steroids for Treatment of C3 Glomerulopathy: A Case Series. *Clin J Am Soc Nephrol* 2018;13(3):406-413. doi: 10.2215/CJN.09080817.

Avasare RS, Canetta PA, Bomback AS, Marasa M, Caliskan Y, Ozluk Y, Li Y, Gharavi AG, Appel GB. Mycophenolate Mofetil in Combination with Steroids for Treatment of C3 Glomerulopathy: A Case Series. Clin J Am Soc Nephrol. 2018;13(3):406-413. doi: 10.2215/CJN.09080817.

Barratt J, Rovin B, Diva U, Mercer A, Komers R; PROTECT Study Design Group. Implementing the Kidney Health Initiative Surrogate Efficacy Endpoint in Patients With IgA Nephropathy (the PROTECT Trial). Kidney Int Rep. 2019 Aug 19;4(11):1633-1637. doi: 10.1016/j.ekir.2019.08.007. PMID: 31891005; PMCID: PMC6933476.

Bernardes TP, Mastroianni-Kirsztajn G. Membranoproliferative glomerulonephritis: current histopathological classification, clinical profile, and kidney outcomes. *J Bras Nefrol*. 2023;45(1):45-50. doi: 10.1590/2175-8239-JBN-2022-0016en

Bomback AS, Smith RJ, Barile GR, et al. Eculizumab for dense deposit disease and C3 glomerulonephritis. *Clin J Am Soc Nephrol* 2012;7(5):748-56. doi: 10.2215/CJN.12901211

Bomback AS, Santoriello D, Avasare RS, et al. C3 glomerulonephritis and dense deposit disease share a similar disease course in a large United States cohort of patients with C3 glomerulopathy. *Kidney Int* 2018;93(4):977-985. doi: 10.1016/j.kint.2017.10.022

Bomback AS, LCH, Yue H, et al. Effect of avacopan, a selective C5a receptor inhibitor, on C3G histologic index of disease chronicity. *J Am Soc Nephrol* 2021;7: S47–S48. https://doi.org/10.1016/j.ekir.2022.01.124

Bomback AS, Appel GB, Gipson DS. Improving Clinical Trials for Anticomplement Therapies in Complement-Mediated Glomerulopathies: Report of a Scientific Workshop Sponsored by the National Kidney Foundation. *Am J Kidney Dis*;2022;79(4):570-581.

Bomback AS, Kavanagh D, Vivarelli M, Meier M, Wang Y, Webb NJA, Trapani AJ, Smith RJH. Alternative Complement Pathway Inhibition With Iptacopan for the Treatment of C3 Glomerulopathy-Study Design of the APPEAR-C3G Trial. *Kidney Int Rep* 2022;7(10):2150-2159. doi: 10.1016/j.ekir.2022.07.004.

Caliskan, Y, Torun, ES, Tiryaki, TO, Oruc, A, Ozluk, Y, Akgul, SU, et al. Immunosuppressive treatment in C3 Glomerulopathy: Is it really effective? *Amer J Nephrol* 2017 46(2), 96–107. https://doi.org/10.1159/000479012

Cameron JS, Turner DR, Heaton J, Williams DG, Ogg CS, Chantler C, Haycock GB, et al. Idiopathic mesangiocapillary glomerulonephritis: comparison of types I and II in children and adults and longterm prognosis. *Amer J Med* 1983;74(2):175-192. doi: 10.1016/0002-9343(83)90606-x

Caravaca-Fontán F, Díaz-Encarnación MM, Lucientes L et al. Mycophenolate Mofetil in C3 Glomerulopathy and Pathogenic Drivers of the Disease. *Clin J Am Soc Nephrol* 2020;15(9):1287-1298. doi: 10.2215/CJN.15241219. Erratum in: *Clin J Am Soc Nephrol* 2020;15(12):1817.

Caravaca-Fontán F, Lucientes L, Cavero T, Praga M. Update on C3 glomerulopathy: a complementmediated disease. *Nephron* 2020;144(6):272-280. doi: 10.1159/000507254

Caravaca-Fontán F, Trujillo H, Alonso M, et al. Validation of a Histologic Scoring Index for C3 Glomerulopathy. *Am J Kidney Dis* 2021;77(5):684-695.e1. doi: 10.1053/j.ajkd.2020.11.011

Caravaca-Fontán F, Díaz-Encarnación M, Cabello V, et al. Longitudinal change in proteinuria and kidney outcomes in C3 glomerulopathy. *Nephrol Dial Transplan*. 2022;37(7):1270-1280. doi: 10.1093/ndt/gfab075

Caravaca-Fontán F, Rivero M, Cavero T, et al. Development and validation of a nomogram to predict kidney survival at baseline in patients with C3 glomerulopathy. *Clin Kidney J* 2022;15(9):1737-1746. doi:10.1093/ckj/sfac108

Caravaca-Fontán F, Praga M. Prognostication for C3 Glomerulopathy and Idiopathic Immunoglobulin-Associated Membranoproliferative Glomerulonephritis. *Clin J Am Soc Nephrol* 2022c Jul;17(7):945-948. doi: 10.2215/CJN.05490522.

Caravaca-Fontán F, Cavero T, Díaz-Encarnación M, et al. Clinical Profiles and Patterns of Kidney Disease Progression in C3 Glomerulopathy. *Kidney360* 2023;4(5):659-672. doi: 10.34067/KID.0000000000000115

Cattran DC, Sethi S (2021). Slowly Unraveling the Mysteries of C3G. *Am J Kidney Dis* 2021;77(5):670-672.

Chauvet S, Hauer JJ, Petitprez F, Rabant M, et al. Results from a nationwide retrospective cohort measure the impact of C3 and soluble C5b-9 levels on kidney outcomes in C3 glomerulopathy. *Kidney Int* 2022;102(4):904-916. doi: 10.1016/j.kint.2022.05.027.

Chen, CH., Wu, HY., Wang, CL. et al. Proteinuria as a Therapeutic Target in Advanced Chronic Kidney Disease: a Retrospective Multicenter Cohort Study. *Sci Rep* 2016;6, 26539.

Cook HT, Pickering MC. Clusters not classifications: making sense of complement-mediated kidney injury. *J Am Soc Nephrol* 2018;29:9-12. doi: 10.1681/ASN.2017111183

Cravedi P, Remuzzi G. Pathophysiology of proteinuria and its value as an outcome measure in chronic kidney disease. *Br J Clin Pharmacol* 2013;76(4):516-523. doi:10.1111/bcp.12104

Damron KC, Friedman R, Inker LA et al. Treating Early-Stage CKD With New Medication Therapies: Results of a CKD Patient Survey Informing the 2020 NKF-FDAScientific Workshop on Clinical Trial Considerations for Developing Treatments for Early Stages of Common, Chronic Kidney Diseases. *Kidney Med*;2022;4(4):100442.

Dixon BP, Greenbaum LA, Huang L, Rajan S, Ke C, Zhang Y, Li L. Clinical Safety and Efficacy of Pegcetacoplan in a Phase 2 Study of Patients with C3 Glomerulopathy and Other Complement-Mediated Glomerular Diseases. *Kidney Int Rep* 2023;8(11):2284-2293. PMID: 38025230

Donadelli R, Pulieri P, Piras R, et al. Unraveling the molecular mechanisms underlying complement dysregulation by nephritic factors in C3G and IC-MPGN. *Front Immunol* 2018;9:2329. doi: 10.3389/fimmu.2018.02329.

Fakhouri F, de Jorge EG, Brune F, Azam P, Cook HT, Pickering MC. Treatment with human complement factor H rapidly reverses renal complement deposition in factor H-deficient mice. *Kidney Int* 2010;78(3):279-86. PMID: 20445496

Fakhouri F, Le Quintrec M, Frémeaux-Bacchi V. Practical management of C3 glomerulopathy and immunoglobulin-mediated MPGN: facts and uncertainties. *Kidney Int* 2020;98(5):1135-1148. doi: 10.1016/j.kint.2020.05.053

Feldman DL, Bomback A, Nester C. Voice of the patient: report of externally-led patient-focused drug development meeting on complement 3 glomerulopathy (C3G). 26 March 2018. Accessed 19 May 2023. https://www.kidney.org/sites/default/files/C3G_EL-PFDD_VoP-Report_3-29-18.pdf

García-de la Puente S, Orozco-Loza IL, Zaltzman-Girshevich S, de Leon Bojorge B. Prognostic factors in children with membranoproliferative glomerulonephritis type I. *Pediatr Nephrol* 2008;23(6):929-935. doi: 10.1007/s00467-008-0754-9

Go AS, Yang J, Tan TC, et al.Contemporary rates and predictors of fast progression of chronic kidney disease in adults with and without diabetes mellitus. *BMC Nephrology* 2018;19(1):146.

Goodship TH, Cook HT, Fakhouri F et al (2017) Atypical hemolytic uremic syndrome and C3 glomerulopathy: conclusions from a "Kidney Disease: Improving Global Outcomes" (KDIGO) Controversies Conference. Kidney Int;91(3):539-551.

Greene T, Ying J, Vonesh EF, Tighiouart H, et al. Performance of GFR Slope as a Surrogate End Point for Kidney Disease Progression in Clinical Trials: A Statistical Simulation. *J Am Soc Nephrol* 2019;30(9):1756-1769. doi: 10.1681/ASN.2019010009. PMID: 31292198; PMCID: PMC6727266.

He P, Zha Y, Liu J, Wang H, He L. Clinical Outcomes of Patients With Primary Membranous Nephropathy and Subnephrotic Proteinuria. *Front Med (Lausanne)*. 2021;8:737700. doi: 10.3389/fmed.2021.737700

Heiderscheit AK, Hauer JJ, Smith RJH. C3 glomerulopathy: Understanding an ultra-rare complement-mediated renal disease. *Am J Med Genet C Semin Med Gene*. 2022;190(3):344-357. PMID: 35734939

Holle J, Berenberg-Goßler L, Wu K, et al. Outcome of membranoproliferative glomerulonephritis and C3-glomerulopathy in children and adolescents. *Pediatr Nephrol* 2018;33(12):2289-2298. doi: 10.1007/s00467-018-4034-z

Hou J, Ren KYM, Haas M (2022). C3 Glomerulopathy: A Review with Emphasis on Ultrastructural Features. *Glomerular Dis* 2022;2(3):107-120.

Hou J, Markowitz GS, Bomback AS, et al. Toward a working definition of C3 glomerulopathy by immunofluorescence. *Kidney Int* 2014;85(2):450-456. doi: 10.1038/ki.2013.340

Huang Z, Wen Q, Zhou SF, Yu XQ. Differential chemokine expression in tubular cells in response to urinary proteins from patients with nephrotic syndrome. *Cytokine* 2008;42(2):222-233. doi:10.1016/j.cyto.2008.02.005

Iatropoulos P, Daina E, Curreri M, et al. Cluster analysis identifies distinct pathogenetic patterns in C3 glomerulopathies/immune complex–mediated membranoproliferative GN. *J Amer Soc Nephrol*. 2018;29(1):283-294. doi: 10.1681/ASN.2017030258

Iatropoulos P, Noris M, Mele C, Piras R, Valoti E, Bresin E, Curreri M, et al. Complement gene variants determine the risk of immunoglobulin-associated MPGN and C3 glomerulopathy and predict long-term renal outcome. *Mol Immunol* 2016;71:131-142. doi: 10.1016/j.molimm.2016.01.010

Inker LA, Mondal H, Greene T, et al. Early Change in Urine Protein as a Surrogate End Point in Studies of IgA Nephropathy: An Individual-Patient Meta-analysis. *Am. J. Kidney Dis* 2016;392-401.

Inker LA, Collier W, Greene T et al. CKD-EPI Clinical Trials Consortium. A meta-analysis of GFR slope as a surrogate endpoint for kidney failure. Nat Med;2023;29(7):1867-1876.

Inker LA, Chaudhari J. GFR slope as a surrogate endpoint for CKD progression in clinical trials. *Curr Opin Nephrol Hypertens*;29(6):581-590.

Kidney Disease: Improving Global Outcomes (KDIGO) Glomerulonephritis Work Group. KDIGO Clinical Practice Guideline for Glomerulonephritis. *Kidney Int*;2012;2:139- 274

KDIGO clinical practice guideline on glomerular diseases – public review draft (2020) https://kdigo.org/wp-content/uploads/2017/02/KDIGO-GN-GL-Public-Review-Draft_1-June2020.pdf Assessed September 12,

Khandelwal P, Bhardwaj S, Singh G, et al. Therapy and outcomes of C3 glomerulopathy and immune-complex membranoproliferative glomerulonephritis. *Pediatr Nephrol* 2021;36(3):591-600. doi: 10.1007/s00467-020-04736-8.

Kovala M, Seppälä M, Räisänen-Sokolowski A, Meri S, Honkanen E, Kaartinen K. Diagnostic and Prognostic Comparison of Immune-Complex-Mediated Membranoproliferative Glomerulonephritis and C3 Glomerulopathy. *Cells*. 2023;12(5):712. doi: 10.3390/cells12050712

Kumar A, Nada R, Ramachandran R, Rawat A, Tiewsoh K, Das R, Rayat CS, et al. Outcome of C3 glomerulopathy patients: largest single-centre experience from South Asia. *J Nephrol* 2020;33(3):539-550. doi: 10.1007/s40620-019-00672-5

Levey AS, de Jong PE, Coresh J, et al. The definition, classification, and prognosis of chronic kidney disease: a KDIGO Controversies Conference report. Kidney Int. 2011 Jul;80(1):17-28. doi: 10.1038/ki.2010.483. Erratum in: *Kidney Int* 2011;80(9):1000. PMID: 21150873.

Levey AS, Gansevoort RT, Coresh J, et al. Change in albuminuria and GFR as end points for clinical trials in early stages of CKD: a scientific workshop sponsored by the National Kidney Foundation in collaboration with the US Food and Drug Administration and European Medicines Agency. *Am J Kidney Dis* 2020;75(1):84-104. doi: 10.1053/j.ajkd.2019.06.009

Lloyd IE, Gallan A, Huston HK, Raphael KL, Miller DV, Revelo MP, Khalighi MA. C3 glomerulopathy in adults: a distinct patient subset showing frequent association with monoclonal gammopathy and poor renal outcome. *Clin Kidney J* 2016;9(6):794-799. doi: 10.1093/ckj/sfw090

Lomax-Browne HJ, Medjeral-Thomas NR, Barbour SJ, et al. Association of Histologic Parameters with Outcome in C3 Glomerulopathy and Idiopathic Immunoglobulin-Associated Membranoproliferative Glomerulonephritis. *Clin J Am Soc Nephrol* 2022;17(7):994-1007. doi: 10.2215/CJN.16801221

Lu DF, Moon M, Lanning LD, McCarthy AM, Smith RJ. Clinical features and outcomes of 98 children and adults with dense deposit disease. *Pediatr Nephrol* 2012;27(5):773-781.
doi: 10.1007/s00467-011-2059-7

Marinozzi MC, Chauvet S, Le Quintrec M, et al. C5 nephritic factors drive the biological phenotype of C3 glomerulopathies. *Kidney Int* 2017;92(5):1232-1241. doi: 10.1016/j.kint.2017.04.017

Mastrangelo A, Serafinelli J, Giani M, Montini G. Clinical and pathophysiological insights into immunological mediated glomerular diseases in childhood. *Front Pediatr* 2020;8:205. doi: 10.3389/fped.2020.00205

Medjeral-Thomas NR, O'Shaughnessy MM, O'Regan JA, Traynor C, Flanagan M, Wong L, Teoh CW, Awan A, Waldron M, Cairns T, O'Kelly P, Dorman AM, Pickering MC, Conlon PJ, Cook HT. C3 glomerulopathy: clinicopathologic features and predictors of outcome. *Clin J Am Soc Nephrol* 2014;9(1):46-53. doi: 10.2215/CJN.04700513. PMID: 24178974; PMCID: PMC3878702.

Mundel P, Reiser J. Proteinuria: an enzymatic disease of the podocyte? *Kidney Int* 2010;77(7):571-80. doi: 10.1038/ki.2009.424. PMID: 19924101; PMCID: PMC4109304.

Nakagawa N, Hasebe N, Hattori M, Nagata M, Yokoyama H, Sato H, Sugiyama H, et al. Clinical features and pathogenesis of membranoproliferative glomerulonephritis: a nationwide analysis of the Japan renal biopsy registry from 2007 to 2015. *Clin Exp Nephrol* 2018;22(4):797-807. doi: 10.1007/s10157-017-1513-7

Nasr SH, Valeri AM, Appel GB, Sherwinter J, Stokes MB, Said SM, Markowitz GS, et al. Dense deposit disease: clinicopathologic study of 32 pediatric and adult patients. *Clin J Amer Soc Nephrol* 2009;4(1):22-32. doi: 10.2215/CJN.03480708

Nester C, Breheny P, Hall M, et al. Relationship between UPCR and eGFR in C3 Glomerulopathy. *Nephrol Dial Transplant* 2021;36(Supp 1):gfab092.0014. doi: 10.1093/ndt/gfab092.0014

Nester C, Appel GB, Bomback AS, et al. Clinical Outcomes of Patients with C3G or IC-MPGN Treated with the Factor D Inhibitor Danicopan: Final Results from Two Phase 2 Studies. *Am J Nephrol* 2022; 53 (10): 687–700. https://doi.org/10.1159/000527167

Nester CM, Eisenberger U, Karras A et al (2022) 12M interim analysis of an open-label, non-randomized extension of phase 2 study to evaluate the long-term efficacy, safety and tolerability of iptacopan in subjects with C3G. American Society of Nephrology. Abstract, control ID: 3764344.

Noris M, Donadelli R, Remuzzi G. Autoimmune abnormalities of the alternative complement pathway in membranoproliferative glomerulonephritis and C3 glomerulopathy. *Pediatr Nephrol* 2019;34(8):1311-1323. doi: 10.1007/s00467-018-3989-0

Okpechi IG, Dlamini TA, Duffield M, et al. Outcome of patients with primary immune-complex type mesangiocapillary glomerulonephritis (MCGN) in Cape Town South Africa. *PloS One* 2014;9(11):e113302. doi: 10.1371/journal.pone.0113302

Paixão-Cavalcante D, López-Trascasa M, Skattum L, et al. Sensitive and specific assays for C3 nephritic factors clarify mechanisms underlying complement dysregulation. *Kidney Int* 2012;82(10):1084-92. doi: 10.1038/ki.

Pickering MC, D'Agati VD, Nester CM, et al. C3 glomerulopathy: Consensus report. *Kidney Int* 2013;84(6):1079–89. doi: 10.1038/ki.2013.377

Pınarbaşı AS, Dursun I , Gokce I et al. Predictors of poor kidney outcome in children with C3 glomerulopathy. *Pediatr Nephrol* 2021 May;36(5):1195-1205. doi: 10.1007/s00467-020-04799-7.

Podos SD, Trachtman H, Appel GB, et al. Baseline clinical characteristics and complement biomarkers of patients with C3 glomerulopathy enrolled in two phase 2 studies investigating the factor D inhibitor danicopan. *Am J Nephrol*;2022;53(10):675-86.

Price CP, Newall RG, Boyd JC. Use of protein:creatinine ratio measurements on random urine samples for prediction of significant proteinuria: a systematic review. *Clin Chem*;2005;51(9):1577-86.

Rabasco C, Cavero T, Román E, et al. Effectiveness of mycophenolate mofetil in C3 glomerulonephritis. *Kidney Int* 2015 Nov;88(5):1153-60. doi: 10.1038/ki.2015.227.

Ravindran A, Fervenza FC, Smith RJ, De Vriese AS, Sethi S. C3 glomerulopathy: ten years' experience at Mayo Clinic. *Mayo Clin Proc* 2018a;93(8):991-1008. doi: 10.1016/j.mayocp.2018.05.019

Schena FP, Esposito P, Rossini M. A narrative review on C3 glomerulopathy: a rare renal disease. *Int J Mol Sci* 2020;21(2):525. doi: 10.3390/ijms21020525

Schmidt T, Afonso S, Perie L et al. An Interdisciplinary Diagnostic Approach to Guide Therapy in C3 Glomerulopathy. *Front Immunol* 2022;13:826513. doi: 10.3389/fimmu.2022.826513. PMID: 35693785; PMCID: PMC9186056.

Schmitt H, Bohle A, Reineke T, et al. Long-term prognosis of membranoproliferative glomerulonephritis type I. *Nephron* 1990;55(3):242-250. doi: 10.1159/000185969

Servais A, Noël LH, Roumenina LT, et al. Acquired and genetic complement abnormalities play a critical role in dense deposit disease and other C3 glomerulopathies. *Kidney Int* 2012;82(4):454-464. doi: 10.1038/ki.2012.63

Sethi S, D'Agati VD, Nast CC, Fogo AB, et al. A proposal for standardized grading of chronic changes in native kidney biopsy specimens. *Kidney Int* 2017;91(4):787-789. doi: 10.1016/j.kint.2017.01.002.

Shabaka A, Cases-Corona C, Fernandez-Juarez G. Therapeutic insights in chronic kidney disease progression. *Front Med (Lausanne)* 2021;8:645187. doi:10.3389/fmed.2021.645187

Simon-Tillaux N, Chauvet S, El Mehdi D, Deschatelets P, Fremeaux-Bacchi V. APL-2 prevents both c3 and c5 convertase formation and activity: a potential therapeutic for renal diseases [Abstract]. *J Am Soc Nephrol* 2019;30:918.

Smith RJ, Appel GB, Blom AM, et al. C3 glomerulopathy—understanding a rare complement-driven renal disease. *Nature Rev Nephrol.* 2019;15(3):129-143. doi: 10.1038/s41581-018-0107-2

Thompson A, Carroll K, Inker LA, et al. Proteinuria Reduction as a Surrogate End Point in Trials of IgA Nephropathy. *Clin J Am Soc Nephrol* 2019;14(3):469-481. doi: 10.2215/CJN.08600718

Thompson A, Smith K, Lawrence J. Change in Estimated GFR and Albuminuria as End Points in Clinical Trials: A Viewpoint From the FDA. *Am J Kidney Dis* 2020;75(1):4-5

Thompson A, Cattran DC, Blank M, et al (2015) Complete and Partial Remission as Surrogate End Points in Membranous Nephropathy. *J Am Soc Nephrol* 2015; 26(12);2930-7.

Troost JP, Trachtman H, Nachman PH, et al. An outcomes-based definition of proteinuria remission in focal segmental glomerulosclerosis. *Clin J Amer Soc Nephrol* 2018;13(3):414.

Viswanathan GK, Nada R, Kumar A et al. Clinico-pathologic spectrum of C3 glomerulopathy-an Indian experience. *Diagn Pathol* 2015;17;10:6.

Wada Y, Kamata M, Miyasaka R et al. Clinico-Pathogenic Similarities and Differences between Infection-Related Glomerulonephritis and C3 Glomerulopathy. *Int J Mol Sci* 2023;8;24(9):8432.

Wong EKS, Hallam TM, Brocklebank V et al. Functional Characterization of Rare Genetic Variants in the N-Terminus of Complement Factor H in aHUS, C3G, and AMD. *Front Immunol*;2021;11:602284.

Wong EKS, Marchbank KJ, Lomax-Browne H et al (2021) MPGN/DDD/C3 Glomerulopathy Rare Disease Group and National Study of MPGN/DDD/C3 Glomerulopathy Investigators: C3 glomerulopathy and related disorders in children: Etiology-phenotype correlation and outcomes. *Clin J Am Soc Nephrol* 2021;16: 1639–51.

VFMCRP and ChemoCentryx provide topline results from ACCOLADE trial of avacopan in C3 glomerulopathy including improved estimated glomerular filtration rate (eGFR) – Vifor Pharma (viforpharmainvestors.com)

Zahir Z, Wani AS, Gupta A, Agrawal V. Pediatric C3 glomerulopathy: A 12-year single-center experience. *Pediatr Nephrol* 2021;36(3):601-610. doi: 10.1007/s00467-020-04768-0

Zhang Y, Nester CM, Martin B, et al. Defining the complement biomarker profile of C3 glomerulopathy. *Clin J Am Soc Nephrol* 2014;9(11):1876-1882. doi: 10.2215/CJN.01820214

Zotta F, Diomedi-Camassei F, Gargiulo A et al (2023) Successful treatment with avacopan (CCX168) in a pediatric patient with C3 glomerulonephritis. *Pediatr Nephrol* 2023 Jun 12. PMID: 37306717.
